# Essential Role of Lyn in Fibrosis

**DOI:** 10.3389/fphys.2016.00387

**Published:** 2016-08-31

**Authors:** Hung Pham, Chiara Birtolo, Chintan Chheda, Wendy Yang, Maria D. Rodriguez, Sandy T. Liu, Gabriele Gugliotta, Michael S. Lewis, Vincenzo Cirulli, Stephen J. Pandol, Andrzej Ptasznik

**Affiliations:** ^1^Division of Gastroenterology, Department of Medicine, Cedars-Sinai Medical CenterLos Angeles, CA, USA; ^2^Department of Veterans AffairsLos Angeles, CA, USA; ^3^Department of Medicine, University of California, Los AngelesLos Angeles, CA, USA; ^4^Department of Internal Medicine, University of BolognaBologna, Italy; ^5^Department of Medicine, Institute of Stem Cell and Regenerative Medicine, University of WashingtonSeattle, WA, USA

**Keywords:** fibrosis, Lyn, Src, pancreatitis

## Abstract

Fibrotic disorders involve replacement of normal parenchyma with myofibroblasts, which deposit connective tissue, leading to obliteration of the function of the underlying organ. The treatment options are inadequate and reflect the fact that signaling targets in myofibroblasts are unknown. Here we identify the hyperactive Lyn signaling in myofibroblasts of patients with chronic pancreatitis-induced fibrosis. Lyn activation coexpress with markers of activated myofibroblasts, and is increased ~11-fold in chronic pancreatitis compared to normal tissue. Inhibition of Lyn with siRNA or INNO-406 leads to the substantial decrease of migration and proliferation of human chronic pancreatitis myofibroblasts *in vitro*, while leaving migration and proliferation of normal myofibroblasts only slightly affected. Furthermore, inhibition of Lyn prevents synthesis of procollagen and collagen in myofibroblasts in a mouse model of chronic pancreatitis-induced fibrosis. We conclude that Lyn, as a positive regulator of myofibroblast migration, proliferation, and collagen production, is a key target for preventing fibrosis.

## Introduction

Fibrosis can occur in many tissues within the body as a result of inflammation or damage. Chronic pancreatitis is an inflammation of the pancreas that leads to fibrotic replacement of the parenchyma, and severe complications, including loss of exocrine and endocrine function, which often leads to secondary diabetes mellitus type 3c (T3cDM) and cancer (Omary et al., 2007). Although it is proven that activated, α-smooth muscle actin (α-SMA)-positive, collagen-producing myofibroblasts (stellate cells), play a key role in infiltration, and replacement of pancreatic parenchyma, no fully effective anti-fibrotic therapies exist to date due to the limited understanding of the key driving signaling mechanisms in these cells (Apte et al., 1998; Omary et al., 2007; Vonlaufen et al., 2010). Parenchyma is also replaced with myofibroblasts and connective tissue during chronic inflammation of liver, and other organs, for which the therapeutic targets are largely unknown, as well. New treatment targets and paradigms are therefore needed.

SDF-1 (stromal cell-derived factor 1) chemokine and its receptor, CXCR4 (Nagasawa et al., 1996), are known as the key regulators of migration of stem/progenitor cells, leukocytes, and other cells, within their local tissue microenvironment. We, and others, have previously shown that Lyn kinase is involved in chemoattractant signaling (Ptasznik et al., 1995, 1996; Mócsai et al., 2000) and is required for CXCR4-mediated chemotaxis of hematopoietic cells and macrophages (Ptasznik et al., 2002; Malik et al., 2008; Tabe et al., 2012). Here we provide results indicating that Lyn is also required for CXCR4-mediated migration of stellate cells in inflamed pancreas.

Lyn has not been linked to fibrotic process in pancreas to date. However, two prior studies have linked Lyn kinase activity to other fibrotic processes. Lyn inhibition attenuates fibrosis in chronic allograft nephropathy models through prevention of plasminogen activator inhibitor-1 (PAI-1), a powerful pro-fibrotic mediator (Pontrelli et al., 2006). Another group suggested that Lyn nitration increases Syk activity and regulates hepatic stellate cell apoptosis in the course of chronic fibrosis of liver (Mòdol et al., 2011). Thus, the mechanisms and consequences are complex and variable. Here we provide evidence that Lyn activity is selectively increased in stellate cells during chronic pancreatitis, which leads to their hyperactive CXCR4-mediated migration, increased proliferation, and excessive collagen production, resulting in chronic fibrosis of pancreas. We suggest that by targeting Lyn, an effective therapy for previously untreatable pancreatic fibrosis may ensue.

## Materials and methods

### Human pancreatic stellate cells

Human stellate cells were examined using freshly harvested pancreatic tissue or freshly isolated primary human stellate cells in culture. Surgical pancreatic samples were obtained from 10 chronic pancreatitis patients, or donors without any pancreatic disease, using guidelines approved by the Institutional Review Board of the Cedars-Sinai Medical Center, Los Angeles, and University of Washington, Seattle. According to the de-identified Surgical Pathology Reports all patients had chronic pancreatitis and fibrotic pancreas, mostly due to intraductal obstructions. Stellate cells were isolated from human pancreatic tissue (by using the tissue digestion method with pronase, collagenase, and DNAse), identified, and expanded in culture, using the same methods as described in detail for rat pancreatic tissue (Apte et al., 1998). In our experiments, we used the early passage cells to minimize any potential effect of prolonged culturing on primary cells. The passage number of chronic pancreatitis or normal cells was always identical for chronic pancreatitis and normal cells during each set of experiment. At 24 h in culture at least 95% of cells had attached to the wells and had assumed a stellate, angular appearance with prominent lipid droplets in the cytoplasm. According to this particular method, purity of the preparation is ultimately ~100% (after a few passages in tissue culture) as assessed by vitamin A autofluorescence in quiescent stellate cells and α–SMA, and procollagen expression in activated stellate cells (myofibroblasts), as earlier described (Apte et al., 1998; Vonlaufen et al., 2010).

### *In vivo* effects of INNO-406 on chronic pancreatitis-induced fibrosis in mouse model

A most commonly used experimental model of chronic pancreatitis-induced fibrosis partially recapitulating human disease is repeated injection of cerulein into mice (Ulmasov et al., 2013). C57BL/6 is the most frequently used mouse strain for this type of biomedical research (Ulmasov et al., 2013). Fibrosis may be reversible only in its early stages. A large body of evidence indicates that established fibrosis results from the replacement of parenchyma with fibrotic tissue and is associated with the massive loss of parenchyma due to necrosis and apoptosis, which in result is irreversible. Therefore, in our current experiments we were focused on preventing fibrosis. Accordingly, C57BL/6J mice (2 months old male and female) were divided into 3 groups (each group consisted of 15 animals) as follows: (1) untreated mice (saline only); (2) mice treated with cerulein for 6 weeks (50 μg/kg hourly, 6 hourly injections in one day constituted one treatment, twice-weekly); (3) mice treated with cerulein (as above) plus 120 mg INNO-406/kg per day for 6 weeks (oral gavage). When 120 mg INNO-406/kg per day is administrated orally to the mice, the concentration of INNO-406 in the soft tissues is estimated to be 0.24 μM according to detailed pharmacokinetic studies described previously (Kimura et al., 2005; Yokota et al., 2007). To allow resolution of acute inflammatory changes (in order to make fibrosis clearly visible and detectable), all mice were euthanized at 5 days after their final cerulein and INNO-406 treatment. Histopathologic pancreas comparison (based on staining with Hematoxylin plus Eosin) confirmed predominantly fibrosis (score 2) with only mild inflammatory infiltrate (score 1) in cerulean-treated mice, in contrast to untreated or cerulean plus INNO-406-treated mice (scores 0). Fibrosis was scored from 0 to 3 (none to extensive fibrosis) and inflammatory infiltrate was scored from 0 to 3 (none to profuse infiltration), as described in detail (Nathan et al., 2010).

### *In vivo* effects of INNO-406 on acute pancreatitis in mouse model

In order to determine whether INNO-406 could potentially prevent the recurrent acute pancreatitis leading to chronic pancreatitis and fibrosis in humans, we investigated the ability of 3 days of pretreatment with INNO-406 to prevent cerulean-induced acute pancreatitis in mice. The primary objective was to test the hypothesis that the proportion of animals with acute pancreatitis following cerulein challenge is significantly lower in animals pre-treated with INNO-406 than in animals pre-treated with placebo. The study was conducted as a randomized, investigator-blinded, controlled, parallel group assessment of the efficacy of INNO-406 in mice with cerulein-induced pancreatitis. *Mice* C57BL/6, sex: male and female, age: 5–6 weeks, weight: ~20–25 g. The study involved dosing 20 mice with INNO-406 (120 mg/kg body weight) or 20 mice with matching placebo (0.5% Carbomethycellulose plus DMSO) by gavage for 3 consecutive days. On the 3rd day, 2 h after gavage, animals received 7 consecutive doses of cerulein (IP, 50 μg/kg body weight) administered at 1-h intervals. At the end of the treatment, on the 3rd day, mice were euthanized and laparotomy was performed and pancreas and blood was collected from euthanized animals for biochemical and histological analysis. *Primary efficacy measure*: proportion of animals with histologic evidence of pancreatitis. Sections of pancreas were stained with hematoxylin and eosin. Reader evaluated 20 high-powered fields and recorded the score for the field with the most severe pathology. *Secondary efficacy measure*: mean pancreas weight/body weight, mean serum amylase, mean serum lipase, mean tissue trypsin, markers of inflammation in pancreas (IL-6, TNF-alpha). The proportions of animals with histologic evidence of pancreatitis in the two treatment groups were compared using Fisher's Exact Test. Other measures were compared using an unpaired *t*-test with Welch correction.

### Immunohistochemistry

The relationship between pancreatic stellate cell activation and pancreatic fibrosis was studied in mice by dual staining of sections for α-SMA plus Picro-Sirius Red for collagen protein (**Figure 3A**). Briefly, frozen tissue sections were fixed with acetone for 10 min at room temperature, stored at −20°C before use and then air-dried for 20 min at room temperature. Sections were washed with PBS three times for 5 min each. Sections were incubated with 0.3% H_2_O_2_ for 10 min to block endogenous peroxidase activity and then washed. To prevent nonspecific binding of antibody, sections were incubated for in a humidified chamber at room temperature for 30 min with a rodent blocking solution (Rodent Block M, Biocare Medical, catalog no. RBM961). Sections were washed again three times for 5 min each and then incubated overnight at 4°C with the anti-alpha SMA primary antibody (mouse monoclonal antibody, clone 1 A4; Sigma-Aldrich, catalog no. A2547), diluted 1:700 in PBS. After further washes, the secondary antibody (Envision+ System-HRP Anti-mouse, Dako, catalog no. K4001) was applied for 1 h at room temperature. After further washes, the color was developed using 3.3-diaminobenzidine tetrahydrochloride (DAB) Peroxidase (HRP) Substrate Kit (Vector Laboratories, catalog no. SK4100). Sections were successively washed with PBS three times for 5 min each. Next, slides were incubated for 20 min with Picro Sirius red solution (Abcam, catalog no. 150681) followed by a brief rinse with acetic acid (0.05%). Sections were dehydrated by washing with absolute alcohol, cleared by washing in Xylene, and covered with synthetic resin (StatLab, Acrymount, catalog no. SL80-4). *Morphometric and statistical analysis*. For the quantification of fibrosis, tissue sections derived from mice of each group (untreated, cerulein-treated, cerulein + INNO-406-treated) were stained with Picro Sirius red solution alone, according to the manufacturing instructions. The slides were digitally scanned at 20X on an Aperio ScanScopAT Turbo (Leica, Buffalo Grove, IL) and stored on Cedars-Sinai server by the Cedars-Sinai Confocal Microscopy Core Facility. Picro Sirius red quantitation was performed using Leica Biosystem Tissue IA Optimizer. For each slide, 15 areas were selected by an investigator, who was unaware of the sample identity, and averaged as a percentage (positive stained area [μm^2^] per total area examined [μm^2^]). Statistical significance of differences in data values was validated by analysis of variance (ANOVA), followed by Sidak's Multiple Comparison Test using the Prism-6 statistical package (Graph Pad Software, San Diego, CA), with significance limit set at *p* < 0.05.

### Immunofluorescence

**Figure 1:**
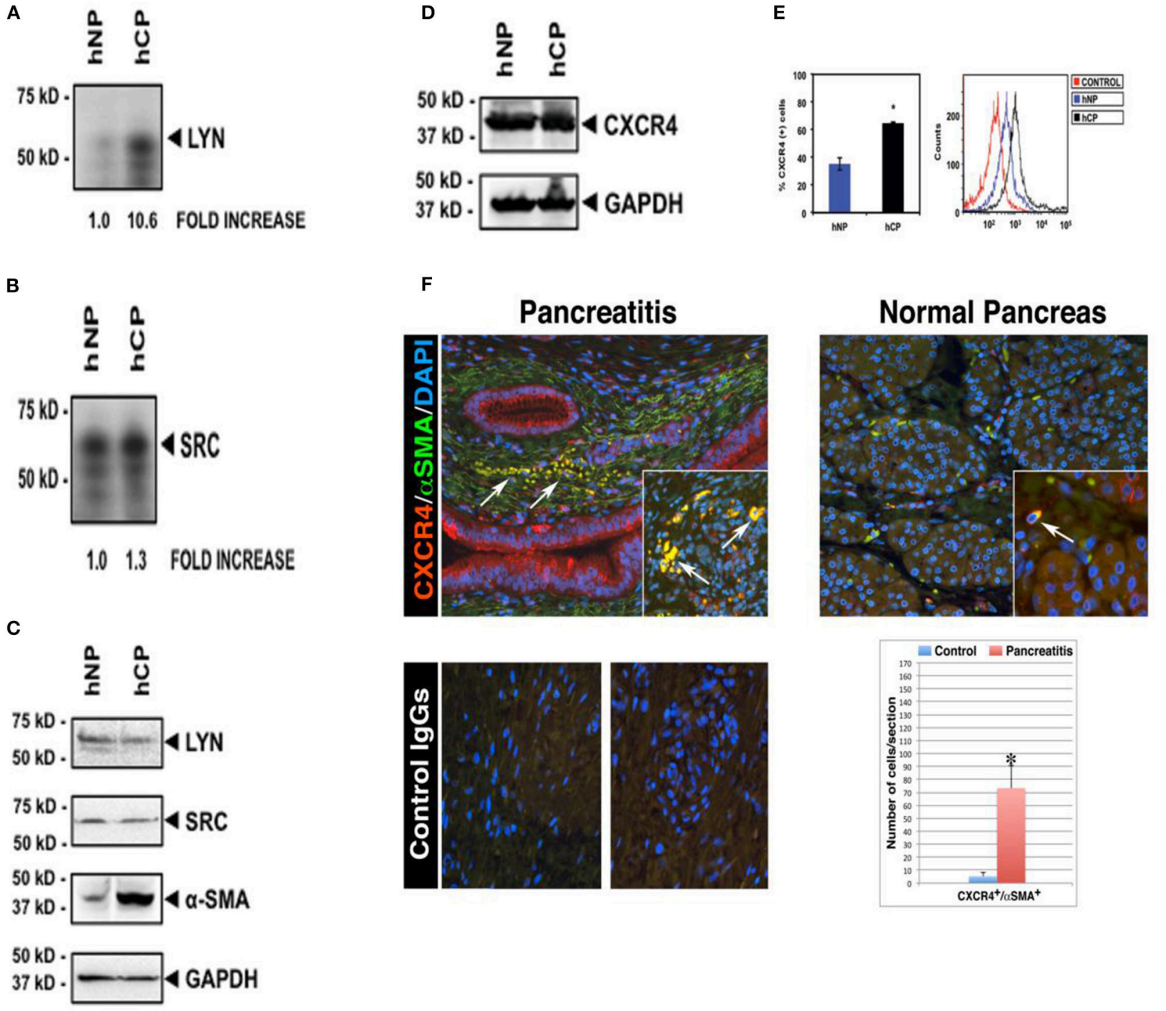
**Increased Lyn kinase activity and CXCR4 receptor expression in activated α-SMA-positive stellate cells in human chronic pancreatitis**. Pancreatic stellate cells were isolated from chronic pancreatitis patients (hCP) and normal donors (hNP). Subsequently, we performed Lyn and Src kinase assays **(A,B)**, and western blottings for Lyn, Src, α-SMA **(C)** and total CXCR4 protein **(D)** in these cells. Kinase assays and Western blot analysis were normalized to the housekeeping gene GAPDH. **(E)** Flow cytometry analysis ofCXCR4 receptor expression on stellate cells of chronic pancreatitis (black) and normal subject (blue). Cells were stained with or without (red) anti-human CXCR4 antibody followed by secondary antibodies and analysis. **(A–E)** are shown for the same pancreatitis and normal subject. We obtained the consistent results with the four different subjects (*n* = 4). **(F)** Representative fields are shown for chronic pancreatitis (*n* = 3) and normal pancreas (*n* = 3). All images shown in this figure are representative of morphometric analysis performed on all human tissue. Bar graph represents the morphometric and statistical analysis performed on 150 images per tissue group and indicates increased coexpression (yellow) of CXCR4 with α-SMA, and infiltration of CXCR4+/α-SMA+ cells into atrophic parenchyma in pancreatitis tissue (arrows, top left quadrant), which is in contrast to the intact parenchymal structures in normal pancreata (top right quadrant). This is consistent with western blot showing increased coexpression of α-SMA with CXCR4 in the pancreatitis patient (hCP) **(C–E)**. For further detail on morphometric and statistical analysis see Section Materials and Methods.

Immunocolocalizations of CXCR4 and α–SMA in pancreatic tissue specimens derived from patients with chronic pancreatitis and normal donors (Figure 1F), or procollagen and α–SMA and F4/80 in pancreatic specimens derived from cerulein-treated or cerulein + INNO-406-treated and untreated control mice (**Figure 3B**), were performed following a method of indirect immunofluorescence with minor modifications, as described (Cirulli et al., 2000; Yebra et al., 2003; Diaferia et al., 2013). Briefly, two and three color immunofluorescence was performed on paraffin sections using standard procedures of deparaffinization followed by antigen retrieval, blocking of aldehyde residues with glycine, and incubation overnight with primary antibodies. Primary antibodies used were: rabbit anti-CXCR4 (Cat#ab2074, Abcam, Cambridge, MA), mouse anti-CXCR4 (Cat# ab58176, Abcam, Cambridge, MA), mouse anti-αSMA (Cat# A2547, Sigma, St. Louis, MO), goat anti-Procollagen-A17 (SC25973, Santa Cruz Biotechnology, Inc., CA), rat anti-F4/80 (Cat# ab6640, Abcam, Cambridge, MA), and mouse anti-E-cadherin (Becton Dickinson, Clone 36/ecad). After primary antibody reaction, sections were challenged with species-specific fluorophore-labeled F(ab)2 secondary antibodies LRSC-donkey anti-rabbit and FITC-donkey anti-mouse, anti-goat, or anti-rat, and LRSC-donkey anti-goat (Jackson ImmunoResearch, West Grove, PA). After staining, sections were mounted with DAPI mounting medium to visualize nuclei, and analyzed at a NIKON Eclipse 90i microscope, equipped with a CoolSNAP-HQ^2^ CCD camera (Photometrics). *Morphometric and statistical analysis*. Extensive morphometric analysis of human tissues was performed on at least 30 sections per tissue samples, collected from three cases of pancreatitis and three normal pancreata, using the NIS Elements 3.22 software (Nikon). Collectively, morphometric analysis of human specimens was performed on 150 images per human tissue group (i.e., pancreatitis or normal pancreas) collected from a total of 180 tissue sections. Morphometric cell counts in mouse tissues were performed on a total of 120 sections (from control mice, and from mice treated with either Cerulein alone, or Cerulein + INNO-406). At least 150 images were acquired *per* animal group. Stained sections were then viewed on a Nikon Eclipse 90i, and morphometric measurement performed using Nikon Pro Plus software (Media Cybernetics, Inc.). Statistical significance of differences in data values was validated by analysis of variance (ANOVA), followed by Bonferroni's Multiple Comparison Test, or by two tailed student's *t*-test, using the Prism-4 statistical package (Graph Pad Software, San Diego, CA), with significance limit set at *p* < 0.05.

### Immune complex kinase assay

Preliminary qPCR experiments determined that only two members of the Src family, Src and Lyn, were substantially expressed in normal and chronic pancreatitis human pancreatic stellate cells. Thus, we performed *in vitro* kinase assays on Src or Lyn immunoprecipitates (Ptasznik et al., 1995, 1996) to measure autophosphorylation (phosphorylation of the kinase by itself sustains its activated state and leads to an increase in enzymatic activity). *Immunoprecipitation*. Aliquots of stellate cells from CP or normal subjects were normalized for cell counts (3.5 × 10^6^ assay), washed in 1 × PBS, and lysed in ice-cold lysis buffer (2% NP-40, 10 mM Tris, 50 mM NaCl, 30 mM sodium pyrophosphate, 50 mM NaF) supplemented with protease inhibitor mixture as described (Ptasznik et al., 1995, 1996). Protein concentrations on whole cell lysates were determined by BCA protein assay. For immunoprecipitation, cell lysates were incubated at 4°C with 5 μl of Lyn or Src Ab for 4 h followed by 30 μl of Pansorbin beads for 2 h. Immunoblot analysis of Lyn, Src, α–SMA, CXCR4, and GAPDH expression, was conducted on immunoprecipitates generated as described above, or directly on cell lysates containing 30 μg of protein. *Kinase assay*. Lyn or Src immunoprecipitates were washed twice with lysis buffer (see above), and once with a buffer containing 10 mM Tris (pH 7.1), 100 mM NaCl, and 0.1 μM sodium orthovanadate. Kinase assays were performed by resuspending Lyn or Src immunoprecipitates in 30 μl kinase reaction mixture (25 mM HEPES [pH 7.1], 10 mM MnCl_2_, 10 μCi [^32^P-γ]–ATP, 1 μM unlabeled ATP). Kinase reaction was carried out for 2 min at 20°C, and then the reaction was stopped by the addition of 30 μl sodium dodecyl sulfate (SDS) gel-loading buffer and boiling, resolved by 8% SDS-PAGE, and visualized by autoradiography.

### siRNA

Four short interfering siRNAs targeting human Lyn and four non-targeting siRNAs (control) combined into one pool (Thermo Scientific, catalog# L-003153-00-0010 and D-001210-01-20, respectively) were prepared and stored frozen in aliquots at −20°C, as described in Supplementary Methods in our previous paper (Ptasznik et al., 2004). A mixture of 4 Lyn siRNAs provided as a single reagent (SMART pool) provides advantages in both potency (reduces Lyn protein by 80–95%) and specificity (very low “off target” silencing effects, as demonstrated by the relevant functional rescue and PKR kinase experiments; Ptasznik et al., 2004). Preparation of primary fibroblast-like stellate cells before electroporation, as well as electroporation with siRNAs, was performed according to the manufacturer's recommendations (for detailed protocols please see the Amaxa 4D-Nucleofector Basic Protocol for Primary Mammalian Fibroblasts and Amaxa 4D-Nucleofector Optimization Protocol for Primary Cells, online).

### Transwell migration assay with SDF-1 in siRNA, AMD3100, and INNO-406-treated cells

SDF-1 was obtained from PeproTech Inc. and AMD3100 from Sigma-Aldrich (St. Louis, MO). INNO-406 was synthetized and purified at Cellagen Technology (San Diego, CA). The migration assays were performed by using Transwell migration assays with polycarbonate membrane inserts with 8 μm pore size that is optimal for fibroblast-like cell migration (Corning Incorporated, NY, cat # 3422). Stellate cells were treated with siRNA (100 nM) for 48 h, or with increasing concentrations of AMD3100 (5–50 μM) or INNO-406 (10–100 nM) for 60 min. Then, cells were placed in the migration upper chambers, in the presence or absence of SDF-1 (0.025 μM) with 2% FBS in the lower chambers. After 2 h of incubation, cells migrated to the lower chamber were counted, and the chemotactic index was determined as follows: (number of cells migrating to SDF-1 chemokine)/(number of cells migrating to medium alone).

### Viability/proliferation measurements

To be certain that unintended cytotoxic effects were not responsible for the observed decreases in cell migration, in siRNA or AMD3100 or INNO-406-treated cells, control viability measurements were carried out by trypan blue exclusion assay (not shown) and MTT assay, during the course of these experiments. As the positive controls for our viability measurements we used stellate cells deprived serum for 24–48 h (not shown). We consistently observed that stellate cells isolated from patients with chronic pancreatitis (hCP) had higher proliferation/viability rate than cells isolated from normal donors (hNP), and Lyn-dependent viability was increased in hCP as compared to hNP. The stellate cell accumulation problem in chronic pancreatitis is a balance equation where: rate of stellate cell accumulation = rate of stellate cell proliferation – rate of stellate cell death. Therefore, we believe that MTT viability assay is the most proper to use in this particular case.

## Results

### Lyn is excessively active in stellate cells of patients with chronic pancreatitis

We began by screening for the activities of known Src family kinases in stellate cells isolated from pancreata of donors with or without chronic pancreatitis (Figure 1). After isolation of stellate cells from human pancreatic tissues, kinase assays showed activities in Lyn and Src immunoprecipitates (Section Materials and Methods). We detected a ~11-fold increase in Lyn kinase activity in cells isolated from chronic pancreatitis compared to cells from normal subjects (Figure 1A). Importantly, we found no increase in Src activity (a kinase of similar structure and function to Lyn; Figure 1B). Thus, increase of Lyn activity in stellate cells of chronic pancreatitis was selective. There was no change in the levels of Lyn or Src proteins, as determined by western blotting (Figure 1C). Importantly, α–SMA, a marker of activation of stellate cells as we described (Omary et al., 2007) was increased in stellate cells from subjects with chronic pancreatitis compared to those from normal subjects (Figure 1C). These data indicated that increased Lyn kinase activity was associated with activation of stellate cells in chronic pancreatitis patients.

### CXCR4(+) stellate cells infiltrate the pancreatic parenchyma of patients with chronic pancreatitis

Next, we investigated the expression of CXCR4 in stellate cells isolated from chronic pancreatitis patients and normal subjects in cultured primary human stellate cells, and also in human pancreatic sections. Western blot analysis of total proteins, normalized to the housekeeping gene GAPDH, showed no differences in the total (surface + intracellular) cellular expression of CXCR4 in stellate cells isolated from chronic pancreatitis patients and normal donors (Figure 1D). In contrast, as indicated by flow cytometry analysis, the mean percentages of stellate cells expressing surface CXCR4 were significantly increased in chronic pancreatitis, as compared to normal cells (Figure 1E). These data indicate that CXCR4 receptor internalization and recycling are altered in chronic pancreatitis stellate cells, as compared to normal stellate cells. Immunocolocalization studies (Section Materials and Methods) indicated the significant colocalization of CXCR4 with α–SMA (marker of activated stellate cells), reaching a ~15-fold increase in chronic pancreatitis tissues, as compared to normal pancreata (Figure 1F). Notably, normal pancreatic tissues exhibited sparse CXCR4-positive activated stellate cells localized between the intact lobular parenchymal structures (top right quadrant in Figure 1F). In sharp contrast, chronic pancreatitis exhibited dense CXCR4-positive activated stellate cells infiltrating atrophic pancreatic parenchyma (see top left quadrant Figure 1F). These results indicated that the number of CXCR4-positive stellate cells, infiltrating the pancreatic parenchyma, was dramatically increased in chronic pancreatitis compared to normal pancreas. All images that are showed in Figure 1F are representative of an extensive morphometric analysis performed on all human tissue (see the bar graph in Figure 1F).

### Differential chemotactic response to stimulation or inhibition of CXCR4/Lyn signaling axis in stellate cells of chronic pancreatitis, as compared to normal donors

**Figure 2:**
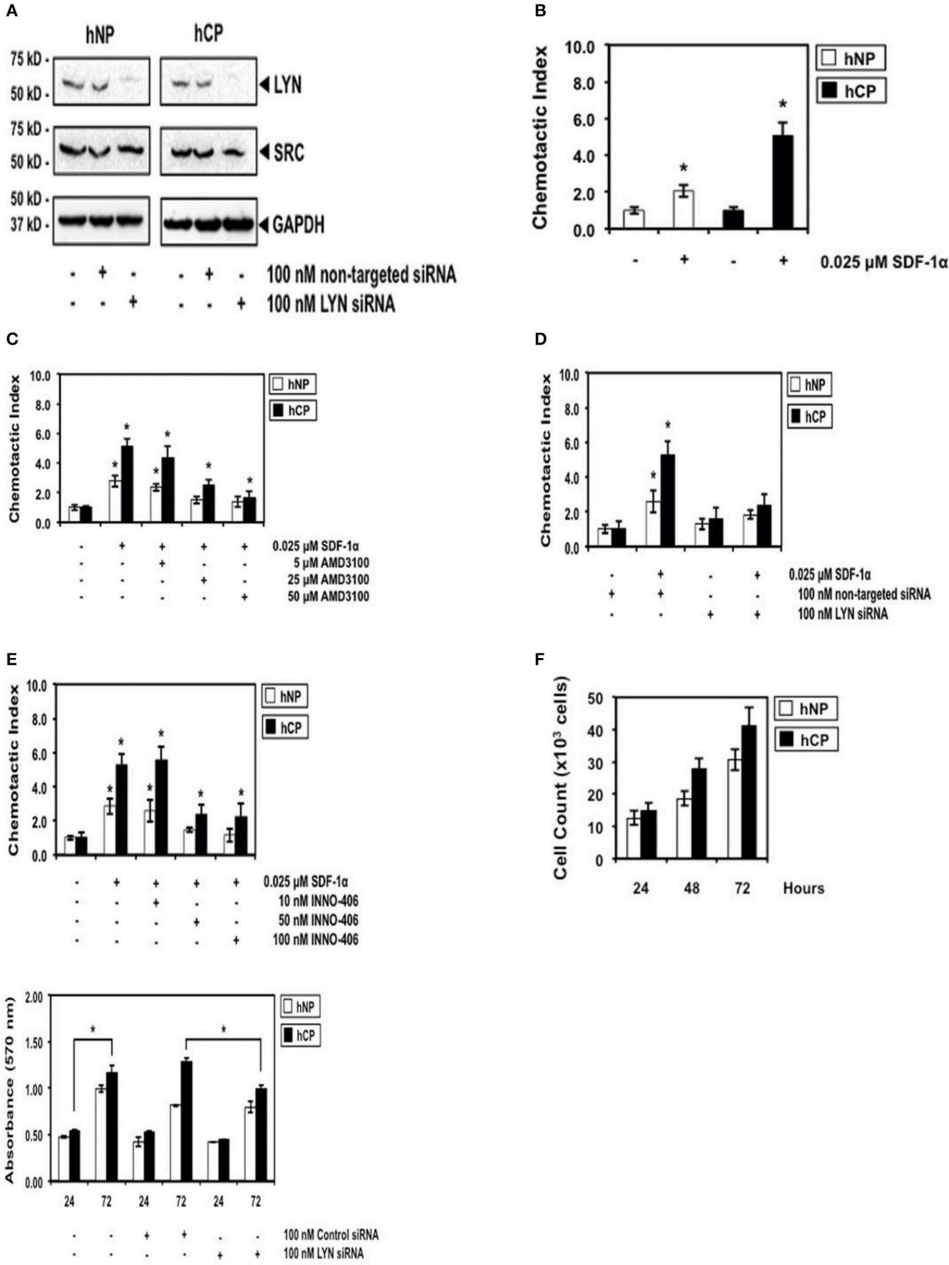
**The robust differential chemotactic response to stimulation or inhibition of CXCR4/Lyn signaling between activated stellate cells of chronic pancreatitis (hCP) and normal donors (hNP). (A)** Control Western blottings were performed 48 h after electroporation with siRNA. **(B)** Cells were stimulated with control buffer or 0.025 μM SDF-1 for 2 h, *n* = 4. **(C–E)** Cells were treated with AMD3100, siRNA, or INN0-406, as indicated, and subsequently we performed chemotaxis assays in SDF-1 stimulated or unstimulated cells (*n* = 4). **(F)** Control cell counts in growing chronic pancreatitis (hCP) vs. normal (hNP) stellate cells, and control MTT viability assays were performed 24 and 72 h after electroporation with siRNA (*n* = 6).

The differential kinase activity of Lyn in pancreatitis stellate cells vs. normal stellate cells (Figure 1A) supports choosing Lyn as a therapeutic target. We silenced Lyn expression in chronic pancreatitis patient-and normal donor-derived stellate cells, using siRNA (Ptasznik et al., 2004). After treatment of the cells (Section Materials and Methods), Western blotting showed a ~80–95% reduction in Lyn protein (Figure 2A). Importantly, we found no inhibitory effect on Src, another Src family kinase of similar structure and size (Figure 2A). Thus, the inhibition of Lyn by siRNA was robust and selective. We also employed a mechanistically distinct approach to inhibit Lyn in stellate cells by using Lyn kinase inhibitor INNO-406 (Bafetinib). INNO-406 was previously shown to be an effective Lyn inhibitor at clinically relevant concentrations without affecting the phosphorylation of Src and several other Src family members (Kimura et al., 2005; Yokota et al., 2007). Thus, INNO-406 is a selective inhibitor of Lyn and not a broad Src family kinase inhibitor.

We then investigated the SDF-1/CXCR4-induced chemotactic response of chronic pancreatitis and normal stellate cells. Chronic pancreatitis cells showed increased CXCR4-induced chemotaxis by ~three-fold as compared to normal cells (Figure 2B), which could be blocked by treatment with AMD3100, a specific antagonist for the SDF-1 receptor CXCR4 (Figure 2C). The selective ablation of Lyn by siRNA (Figure 2A) effectively inhibited chemotaxis of pancreatitis stellate cells (Figure 2D). We also observed strong inhibition of chemotaxis following treatment with INNO-406 (Figure 2E). Notably, inhibition of chemotaxis by Lyn inhibitors (siRNA or INNO-406) was much stronger in stellate chronic pancreatitis cells than in normal cells (Figures 2D,E), consistent with differential Lyn kinase activity (Figure 1A). In addition, the selective ablation of Lyn by siRNA (Figure 2A) effectively inhibited viability of chronic pancreatitis stellate cells at 72 h, while leaving viability of normal stellate cells not affected (Figure 2F). These findings indicated that Lyn stimulated both chemotaxis and viability of stellate cells (this function of Lyn was previously established in other cellular systems; O'Laughlin-Brunner et al., 2001; Ptasznik et al., 2002) and Lyn-mediated chemotaxis and viability of stellate cells were increased in chronic pancreatitis, consistent with hyperactive Lyn kinase in chronic pancreatitis (Figure 1A).

### Lyn is a key target for preventing chronic pancreatitis-induced fibrosis

Our findings with human primary stellate cells support targeting Lyn in chronic pancreatitis patients by showing that Lyn activity is differentially expressed in stellate cells of chronic pancreatitis compared to normal subjects, and inhibition of Lyn leads to the significant chemotaxis and proliferation decrease of primary chronic pancreatitis stellate cells *in vitro*, while leaving chemotaxis and proliferation of normal stellate cells only slightly affected (Figures 1, 2). Although it is known that selective inhibition of one of the many chemokine receptors can be easily bypassed *in vivo* through other chemokine receptors in chronic inflammation, including chronic pancreatitis, targeting a common downstream intracellular target may inhibit the bypass effect. This is important because the mean percentages of stellate cells expressing surface CCR5 (in addition to CXCR4) were significantly increased in chronic pancreatitis as compared to normal stellate cells (not shown), and CCR5 mediates Lyn signaling in other cellular systems. Inhibition of Lyn would therefore be difficult to bypass *in vivo*, because Lyn is a common downstream target of several key pro-inflammatory chemoattractant receptors, including CXCR4, CCR5, and fMLP receptor (Ptasznik et al., 1995, 1996, 2002; Mócsai et al., 2000; Nakata et al., 2006; Tomkowicz et al., 2006; Malik et al., 2008; Tabe et al., 2012; Hu et al., 2014). Thus, inhibition of Lyn prevents the chemotactic and proliferative action of several receptors in different cellular systems (Ptasznik et al., 1995, 1996, 2002; Malik et al., 2008; Tabe et al., 2012).

**Figure 3:**
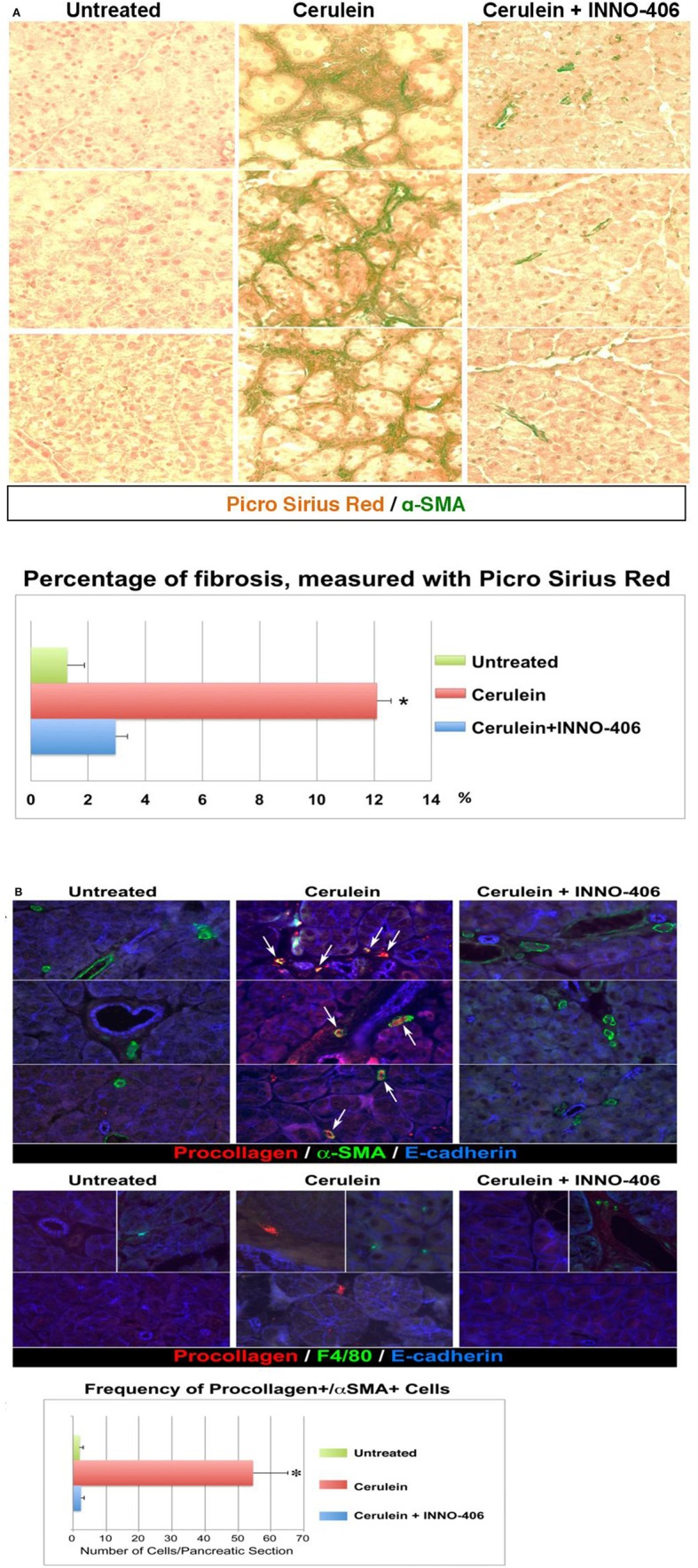
**Pancreatic fibrosis is prevented by treatment with Lyn kinase inhibitor, INN0-406, in a cerulein-induced mouse model of chronic pancreatitis. (A)** Dual staining (Picro-Sirius Red plus a-SMA) denotes fibrosis: collagen (dark red) with adjacent a-SMA positive activated myofibroblasts (green). **(B)** Immunofluorescence, increased frequency of procollagen, and a-SMA-positive cells in mice with chronic pancreatitis. Procollagen and α-SMA coexpression in myofibroblasts is highlighted by the appearance of a yellow color (arrows) resulting from the overlap of red and green. The frequency of cells coexpressing procollagen and a-SMA is substantially decreased in INN0-406-treated mice. Note that the F4/80-positive macrophages (green, middle panel) do not exhibit procollagen-specific immunoreactivity, and are not adjacent to myofibroblasts expressing procollagen (red). All images shown in this figure are representative of an extensive morphometric analysis performed on the all mice pancreatic tissue (see the bar graphs). For detail on morphometric and statistical analysis see Section Materials and Methods.

We therefore performed *in vivo* validation of Lyn as a single therapeutic target in myofibroblasts of chronic pancreatitis-induced fibrosis (Section Materials and Methods). The all images that are showed in our paper are representative of an extensive morphometric analysis performed on the mice pancreatic tissue (see the bar graphs in Figure 3). Dual staining approaches revealed fibrosis and a strong association between fibrosis and activated stellate cells in pancreas of cerulein-treated mice (Figure 3A). In the cerulein-treated mice, Sirius Red collagen staining was intense and collagen was adjacent to α–SMA-positive myofibroblastic stellate cells (Figure 3A). The extent of fibrosis was substantially reduced in mice treated for 6 weeks with both cerulein and Lyn kinase inhibitor INNO-406 and there was less collagen adjacent to α–SMA-positive stellate cells in these mice (Figure 3A). Immunocolocalization studies detected numerous activated stellate cells coexpressing procollagen and α–SMA in pancreata from cerulean-treated mice, as compared to untreated mice, reaching a ~30-fold increase (Figure 3B). Treatment of mice with INNO-406, in conjunction with cerulein, resulted in a drastic reduction of procollagen-specific immunoreactivity in α–SMA-positive stellate cells (Figure 3B). In contrast, F4/80-positive macrophages identified in all groups of mice did not exhibit procollagen-specific immunoreactivity, as expected, and were not adjacent to activated stellate cells expressing procollagen (Figure 3B). These results strongly indicate that activated stellate cells co-localize with areas of chronic pancreatic-induced fibrosis and are the source of Lyn-mediated procollagen/collagen production.

## Discussion

We show that the ultimate results of Lyn inhibition *in vivo* is reduced fibrosis (Figure 3), but the mechanism is still unclear. The most likely mechanism for the inhibition of fibrosis *in vivo* we have just described is suggested by observations that INNO-406 and Lyn siRNA directly prevent chemokine-induced cell movement, proliferation, and viability in purified activated stellate cells *in vitro* (Figure 2). We propose that the high Lyn kinase activity in activated stellate cells (Figure 1A) drives invasiveness and proliferation of these cells in chronic pancreatitis. Therefore, the direct and selective inhibition of Lyn kinase (using INNO-406 or siRNA) could prevent expansion and infiltration of these fibroblastic cells and thus fibrosis. Alternatively, it is possible that, *in vivo*, Lyn inhibition results in reduced fibrosis by attenuating inflammatory tissue damage (e.g., by inhibition of inflammatory infiltration). Therefore, we also examined whether INNO-406 could prevent acute pancreatitis in a cerulein-induced pancreatitis mouse model (Section Materials and Methods). We determined that there was no effect of INNO-406 on the acute pancreatitis and inflammatory tissue injury (not shown), ruling out inhibition of inflammation as a major cause of reduced fibrosis in INNO-406-treated mice (Section Materials and Methods). These findings suggest that the selective Lyn inhibition results in reduced fibrosis by attenuating movement, infiltration, proliferation, viability (Figure 2), and collagen production (Figure 3) in stellate cells rather than by direct attenuating pancreatitis and inflammatory tissue damage.

On first examination our present results seem to be in contrast to our previous data indicating critical role of Src kinases, including Lyn, in acute inflammation in general (Ptasznik et al., 1995, 1996; Liu et al., 2013). However, since Lyn is also directly associated with invasive cell phenotype, including aberrant cell movement, infiltration and prolonged viability (Ptasznik et al., 2002, 2004; Nakata et al., 2006; Malik et al., 2008), it is possible that the hyperactive Lyn (Figure 1A) is simply driving invasiveness of activated myofibroblasts in pancreatitis, leading ultimately to fibrosis. The other authors have recently used the broad inhibitor of kinases, PP2, and concluded that Src family kinases are the key mediators of inflammation and acute pancreatitis (Nuche-Berenguer et al., 2016). However, despite common use of PP2 as a “selective” inhibitor of Src-family kinases, extensive research has shown that this particular compound is non-selective and blocks many other kinases with similar affinities (Brandvold et al., 2012). On the other hand, the broad kinase inhibitor, PP2, could prevent redundancy of Src family kinases and other kinases and thus be more effective in inhibiting acute pancreatitis than the selective Lyn inhibitor, INNO-406. In any case, we conclude here that the exact biochemical mechanism for the involvement of Lyn in pancreatitis-induced fibrosis is still unknown and this is the legitimate object of additional studies.

Our studies uncover an essential role of the Src family kinase Lyn in the development of pancreatic fibrosis. Interestingly, a role for Src family kinases in activated myofibroblasts was recently reported in pulmonary fibrosis (Hu et al., 2014). However, this earlier study (Hu et al., 2014) was not designed to specifically address the individual roles of any specific Src kinase member in activated myofibroblasts. In the present paper, we use specific Lyn kinase inhibitors (siRNA or INNO-406) and discover a role of the Src family kinase Lyn as a positive regulator of activated myofibroblast migration, proliferation, and collagen production, which are all characteristics of fibrosis. We conclude that Lyn is an emerging relevant target in anti-fibrotic therapeutic strategies, and the inhibition of Lyn kinase in stellate cells prevents the replacement of parenchyma with fibrotic tissue in pancreas. Since fibrotic process, which result from myofibroblast activation, can occur in many tissues within the body, the role of Lyn might be a general phenomenon and relevant in other tissues besides pancreas, as it was previously suggested by other authors (Pontrelli et al., 2006; Mòdol et al., 2011). Notably, reactive nitrogen species generated in inflammation can create nitrated biomolecules in various tissues and the role of tyrosine nitration in the mechanism of stable Lyn tyrosine kinase activation was previously reported in other tissues besides pancreas (Mallozzi et al., 2001). This would explain why cultured stellate cells of chronic pancreatitis patients sustain their biological behavior at early passage *in vitro*, for example, the very high Lyn kinase activity (Figure 1A). Moreover, Lyn-mediated pathways might also be potentially relevant to tissue injury or radiation therapy-induced fibrosis (Leung et al., 2002), which are likely associated with activation of myofibroblasts. Future studies directed at the upstream and downstream signaling elements coupled to Lyn should prove informative, as will investigation of the transcriptional factors by which Lyn links to collagen-related gene regulation in activated myofibroblasts in pancreas and other tissues.

## Author contributions

Conception and design of research: AP, SP. Performed experiments: HP, CB, CC, WY, MR, SL, GG, ML, VC, AP. Edited manuscript: AP, SP.

### Conflict of interest statement

The authors declare that the research was conducted in the absence of any commercial or financial relationships that could be construed as a potential conflict of interest.

## Abbreviations

α-SMA: alpha smooth muscle actin
CXCR4: chemokine (C-X-C motif) receptor 4
F4/80: a mature mouse cell surface glycoprotein expressed at high levels on various macrophages
INNO-406, Bafetinib: a potent and selective Lyn inhibitor
Lyn, LYN proto-oncogene: Src family protein tyrosine kinase
PAI-1: plasminogen activator inhibitor-1
PP2 (kinase inhibitor): 4-amino-5-(4-chlorophenyl)-7-(dimethylethyl)pyrazolo[3,4-*d*]pyrimidine
SDF-1: stromal-cell derived factor-1, chemokine
siRNA: small interfering RNA, a synthetic RNA duplex designed to specifically target a particular mRNA for degradation
Syk: spleen tyrosine kinase, non-receptor type Tyr protein kinase
Src: SRC proto-oncogene, non-receptor tyrosine kinase.
